# Low-phosphorus concentrations and important ferric hydroxide scavenging in Archean seawater

**DOI:** 10.1093/pnasnexus/pgad025

**Published:** 2023-02-08

**Authors:** Eric Siciliano Rego, Vincent Busigny, Stefan V Lalonde, Camille Rossignol, Marly Babinski, Pascal Philippot

**Affiliations:** Instituto de Geociências, Departamento de Mineralogia e Geotectônica, Universidade de São Paulo, Rua do Lago 562, Cidade Universitária, São Paulo, 05508-080, Brasil; Géosciences Montpellier, Pl. Eugène Bataillon, Campus Triolet, Université de Montpellier, CNRS, Université des Antilles, 34095, Montpellier, France; Université Paris Cité, Institut de physique du globe de Paris, CNRS, 1 Rue Jussieu, 75238 Paris cedex 05, France; CNRS-UMR6538 Laboratoire Geo-Ocean, Institut Universitaire Européen de la Mer, Université de Bretagne Occidentale, Technopôle Brest-Iroise, Rue Dumont d'Urville, 29280, Plouzané, France; Dipartimento di Scienze Chimiche e Geologiche, Università degli Studi di Cagliari, Str. interna Policlinico Universitario, 09042 Monserrato, Cagliari, Italia; Instituto de Geociências, Departamento de Mineralogia e Geotectônica, Universidade de São Paulo, Rua do Lago 562, Cidade Universitária, São Paulo, 05508-080, Brasil; Géosciences Montpellier, Pl. Eugène Bataillon, Campus Triolet, Université de Montpellier, CNRS, Université des Antilles, 34095, Montpellier, France; Departamento de Geofísica, Instituto de Astronomia, Geofísica e Ciências Atmosféricas, Universidade de São Paulo, Rua do Matão 1226, Cidade Universitária, São Paulo, 05508-090, Brasil

**Keywords:** phosphorus, Archean, iron formations

## Abstract

The availability of nutrients in seawater, such as dissolved phosphorus (P), is thought to have regulated the evolution and activity of microbial life in Earth's early oceans. Marine concentrations of bioavailable phosphorus spanning the Archean Eon remain a topic of debate, with variable estimates indicating either low (0.04 to 0.13 μM P) or high (10 to 100 μM P) dissolved P in seawater. The large uncertainty on these estimates reflects in part a lack of clear proxy signals recorded in sedimentary rocks. Contrary to some recent views, we show here that iron formations (IFs) are reliable recorders of past phosphorus concentrations and preserved a primary seawater signature. Using measured P and iron (Fe) contents in Neoarchean IF from Carajás (Brazil), we demonstrate for the first time a clear partitioning coefficient relationship in the P-Fe systematics of this IF, which, in combination with experimental and Archean literature data, permits us to constrain Archean seawater to a mean value of 0.063 ± 0.05 μM dissolved phosphorus. Our data set suggests that low-phosphorus conditions prevailed throughout the first half of Earth's history, likely as the result of limited continental emergence and marine P removal by iron oxyhydroxide precipitation, supporting prior suggestions that changes in ancient marine P availability at the end of the Archean modulated marine productivity, and ultimately, the redox state of Earth's early oceans and atmosphere.

**Classification:** Physical Sciences, Earth, Atmospheric and Planetary Sciences

**Significance Statement**

The evolution of life in Earth's oceans is thought to have been dependent on nutrient availability, such as dissolved phosphorus (P) in seawater. Quantitatively assessing P concentrations in ancient oceans, however, is challenging and a highly debated topic. Here, we report a correlation between P and Fe contents measured in 2.74 billion-year-old iron formations and carbonates showing the same characteristics as those recorded in modern sediments. We further show that, similarly to present-day environments, Fe-oxide adsorption was a major exit flux of phosphorus from seawater. Our results, combined with available experimental data from the literature, argue for strongly limited P contents on early Earth.

## Introduction

Phosphorus (P) is an essential nutrient for all living organisms on Earth and its availability has been crucial for the development and proliferation of life on geological time scales (1). In the oceans, P availability has a significant control on biological productivity, and its concentration throughout Earth's history likely modulated the redox state of the ocean and atmosphere (2–4). Quantitatively assessing past oceanic P levels based on the geological record has been a major challenge, largely due to a lack of robust mineralogical or geochemical proxies that provide unambiguous records of water column P concentrations. Despite the challenge posed by the limited sedimentary record, understanding the ancient marine P cycle is of paramount importance for understanding biological and geochemical evolution through time (5, 6).

Reconstructions of dissolved P concentrations in Precambrian oceans are highly debated and rely to a large extent on iron (Fe) and P contents measured in iron-rich chemical sediments, which are interpreted to reflect seawater conditions at their time of formation (2, 5, 7). Previous works support contrasting views of either low dissolved P concentrations in Archean seawater (<1 μM) compared with average modern values (∼2.3 μM) (2, 7), or a significantly higher P content in seawater, particularly prior to 2.45 Ga, with proposed concentrations ranging from 5 to 50 times higher than modern values (∼10 to 100 μM) (8, 9).

Phosphorus concentrations in ancient seawater can be estimated using the phosphorus to iron ratio (P/Fe) in well-preserved Fe-rich chemical sedimentary rocks (2, 5, 7, 10). This relies on the premise that iron oxide P/Fe ratios scale linearly as a function of ambient dissolved phosphate (PO_4_^3−^) concentrations according to a predictable distribution coefficient model, as observed to occur in modern hydrothermal plumes (11). In this approach, dissolved phosphate concentration (*P*_D_) in a given solution is equal to the P/Fe ratios in ferric oxyhydroxide precipitates divided by a specific distribution coefficient (*K*_D_) representative of the fluid's composition, such that [*P*_D_] = (1/*K*_D_) × P/Fe (2). Given that *K*_D_ values will change in relation to the fluid's composition, as shown by the enrichment and/or depletion of Si and divalent cations (Ca^2+^, Mg^2+^) inhibiting and/or facilitating P adsorption, experimental studies have proposed distribution coefficients representative of Archean and Proterozoic seawater (7, 10), thus allowing new estimates of dissolved P in ancient ocean to be determined.

However, despite a clear correlation between P and Fe in modern sediments (11, 12), no such evidence has ever been reported in samples from the Precambrian rock record. In contrast, a lack of correlation was observed in ∼2.45 Ga old iron formations (IFs) from Australia and South Africa, suggesting that these rocks did not capture past oceanic conditions (8). It remains to be seen whether other IF deposits may record P-Fe systematics indicative of P uptake behavior conforming to the distribution coefficient model and thus may be useful for constraining the paleomarine P reservoir. Here, we use P and Fe contents from Archean iron-rich chemical sediments to investigate the potential of IFs as recorders of past P contents in seawater and to estimate dissolved P concentrations in seawater prior to the great oxidation event (GOE). Our work is based on Neoarchean (∼2.74 Ga) sedimentary rocks from Carajás, Brazil, along with previously published P/Fe ratios from Archean IFs, modern environmental data, and laboratory experiments. The sedimentary succession studied (see Figs. S1–S3) includes IFs intercalated with several Fe-rich and Fe-poor carbonate intervals, therefore representing an opportunity for evaluating P levels in the ocean independently of these lithological variations. Here, we demonstrate a clear example from the Archean sedimentary record of the positive P-Fe scaling relationship expected from P adsorption to Fe oxide, confirming the predominance of Fe oxide as a P sink in these samples and firmly validating the distribution coefficient approach for reconstruction of the ancient marine P reservoir. Collectively, the results provide key insights into past nutrient availability, which likely influenced the dynamics of oxygen production and accumulation in Earth's Precambrian oceans and atmosphere.

## Results and discussion

### Identifying an Archean Fe-P trap

**Fig. 1.**
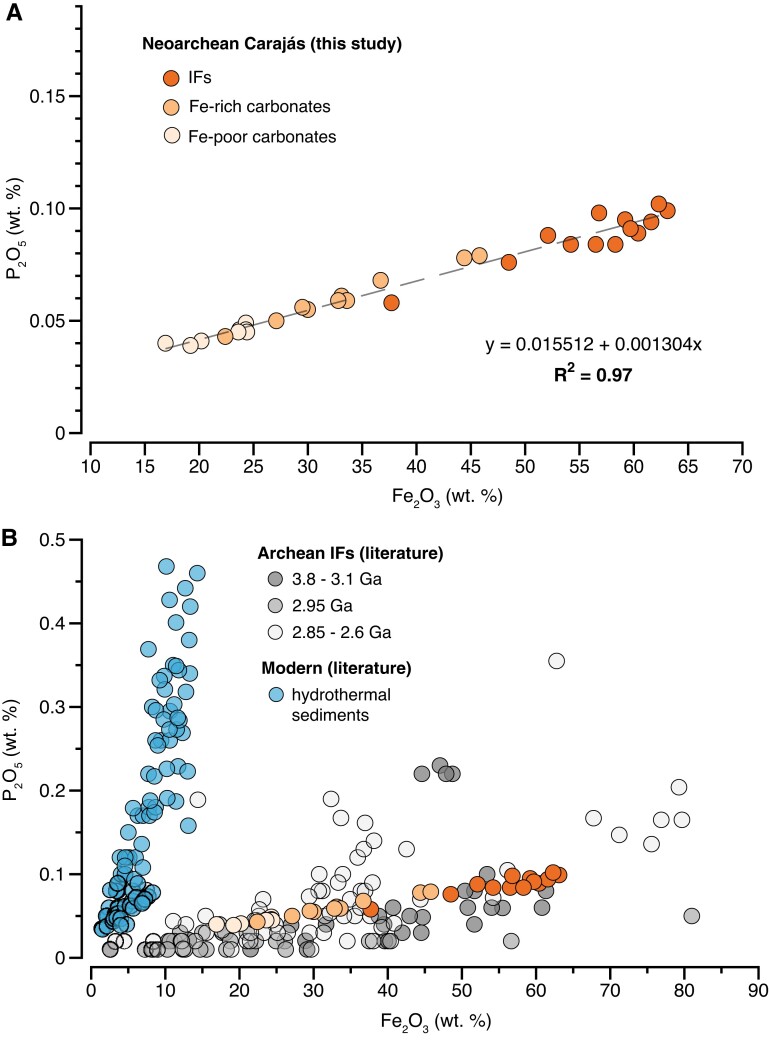
Variations of bulk Fe_2_O_3_ and P_2_O_5_ measured in (A) Neoarchean (∼2.74 Ga old) iron formations, Fe-rich and Fe-poor carbonates from Carajás Brazil, and compared in (B) with Archean IF literature data (3.8–2.6 Ga; 2, 5, 16–22) and modern hydrothermal sediments (23).

The weight percent (wt.%) variations of P_2_O_5_ and total Fe (Fe_2_O_3_) in the studied IF samples range from 0.076 to 0.1 wt.% and 37.7 to 63.1 wt.%, respectively, while Fe-carbonates (Fe-rich and Fe-poor) have P_2_O_5_ and Fe_2_O_3_ contents varying between 0.039 and 0.079 wt.% and between 16.9 and 45.8 wt.%, respectively (Table S1). A well-defined positive correlation is observed between the concentrations of P_2_O_5_ and Fe_2_O_3_ measured in the different chemical sedimentary lithologies from the Carajás Formation (Fig. 1A). In contrast, P_2_O_5_ shows rough inverse relationships with CaO, MgO, and MnO contents (Fig. S4), indicating that iron is the main ligand of phosphorus and that carbonate-rich samples contain less phosphorus. Aluminum is a very minor component in Carajas samples (Al_2_O_3_ < 0.1 wt%) and shows no correlation with phosphorus content (Fig. S4). Moreover, when compared with whole-rock IF literature data filtered for low detritus (<0.3 wt.% Al), our samples are within the range of values measured for other Archean samples (3.8 to 2.6 Ga, Fig. 1B), although less scattered. Our results show a similar trend to the one measured in modern hydrothermal systems (e.g. positive correlation between P_2_O_5_ and Fe_2_O_3_) but with a different slope. The modern correlation is interpreted as reflecting the adsorption of dissolved bioavailable phosphate (PO_4_^3−^) onto Fe-oxide precipitates (11–13). Previous studies relied on this observational model to propose an active P-removal from seawater by Fe-oxide precipitation in the Archean ocean (Fe-P trap; 2, 7). Potential phosphite species that may have been generated during sedimentary diagenesis (e.g. 14) are ignored, as these are negligible in the modern marine P budget, unreported at present for rocks deposited after 3.5 Ga (15) and counter indicated in our sample set by the consistent relationship between P and Fe.

The trend defined by the Carajás samples consisting of different rock types (carbonates and IFs) further supports the interpretation that Fe-oxides were likely responsible for P removal from seawater during the Archean, as observed in modern environments (Fig. 1). This, in turn, indicates that ferric oxyhydroxides, and not simply ferrous hydroxides, were primary mineral phases in IFs (e.g. 24). More importantly, our data demonstrate empirically for the first time that IFs may indeed show P-Fe systematics consistent with aqueous adsorptive and co-precipitative partitioning according to the distribution coefficient model. This evidence confirms that a Fe-P trap mechanism was in fact operating, certainly for the Carajás IF (2.74 Ga), and probably for others deposited between 3.8 and 2.6 Ga. Whether this mechanism limited past ocean P availability (e.g. dissolved P concentrations) is still an ongoing question (8) and requires further assessment. Nonetheless, given the correlation between P and Fe for the Carajás samples (*R*^2^ = 0.97), we can estimate the concentration of dissolved P in seawater using the slope of the linear regression (∼0.0013) combined with previous published distribution coefficient ([*K*_D_]) values (Tables 1 and S2).

**Table 1.** Estimations of dissolved phosphorus for Carajás based on *K*_D_ values taken from adsorption experiments with seawater.

Experimental conditions	*K*_D_ values	P (μM)^a^
Seawater + 0 mM Si	0.338	0.004
Seawater + 0.67 mM Si	0.042	0.03
Seawater + 2.2 mM Si	0.008	0.18

a Estimated dissolved P based on P/Fe mol ratio (0.0015) for Carajás IFs.

### Archean P vs. Fe trend—preservation vs. alteration

**Fig. 2.**
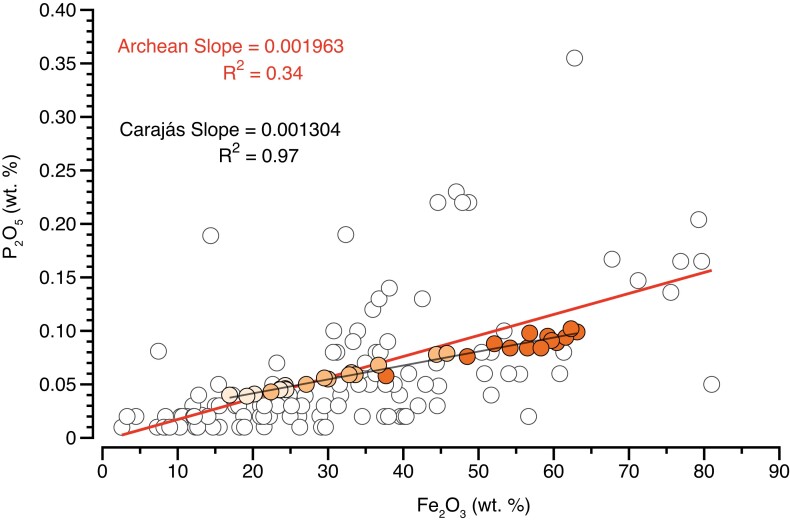
Comparison of linear regressions based on Fe_2_O_3_ (wt. %) and P_2_O_5_ (wt. %) contents from Archean and Carajás data sets showing their respective slopes and *R*^2^ coefficients.

The Archean literature data set broadly overlaps with the newly analyzed Carajás samples. However, literature values display larger scattering in P-Fe space (*R*^2^ = 0.34), which contrasts with well-correlated Carajás values (e.g. *R*^2^ = 0.97; Fig. 2). Such differences could be related to rock preservation effects. For instance, a loss of Fe and/or P during diagenetic and metamorphic reactions would be expected to produce some variability, hence scattering, in the primary composition of the sediments. These conditions are observed in the ancient rock record, where Fe can be leached by metasomatic fluids (25), and also in modern environments analogous of Archean settings, where the reduction and dissolution of ferric oxyhydroxides release previously adsorbed phosphate to the water column, allowing Fe(II) phosphate mineral (e.g. vivianite) to precipitate (26). Vivianite, once precipitated, can react with calcite with increasing temperature and pressure to form apatite (3). Considering that a reduction of Fe(III) minerals was likely occurring in the Archean (e.g. dissimilatory iron reduction) (16, 27, 28) and that each location was exposed to different metamorphic regimes (e.g. low-greenschist to high-amphibolite facies), it is likely that varying proportions of Fe and P loss will be imprinted in Archean samples, thus supporting the scattering observed in Fig. 2.

In contrast, the preserved correlation between Fe and P in Neoarchean Carajás samples can be explained by two scenarios. These are (i) minimal loss of Fe and P, suggesting remarkable sample preservation, resembling modern hydrothermal environments and/or (ii) loss of Fe and P in similar molar proportions, which would not have affected the primary correlation. The first scenario might have been favored for the Carajás IF considering that it shows abundant carbonate-facies IF, and that the same enhanced alkalinity favoring calcium carbonate precipitation may also act to trap P in sediments by promoting carbonate fluorapatite formation (17). Although the second scenario seems unlikely, we cannot completely exclude it. In any case, this is not critical to our interpretation as it would imply an unmodified P/Fe ratio. Moreover, measured P/Fe ratios from Neoarchean Carajás and Archean literature data both define a log normal distribution (Fig. S5), while P and Fe concentrations show linear correlations with slopes of 0.0013 (*R*^2^ = 0.97) for Carajás and 0.0020 (*R*^2^ = 0.34) for Archean data (Fig. 2). The P/Fe ratio of Carajás IF is thus not significantly different from that of Archean IFs and it seems reasonable to consider Carajás as a representative but best preserved. Therefore, estimates of dissolved P based on P/Fe ratios in the Archean (e.g. 5, 7) should be well-represented by combining and comparing the two data sets.

### Phosphorous-limited Archean ocean

Laboratory experiments have demonstrated that Si and other ferrihydrite-reactive ions may significantly influence P adsorption by competitive interactions, especially Si (10). Given that Archean oceans had a different chemistry compared with modern oceans, Jones et al. (7) proposed a wide range of distribution coefficients (K_D_) representative of Precambrian's seawater composition. When combining all K_D_ values for solutions with varying Si, Ca^2+^, and Mg^2+^ contents with the P/Fe ratio (0.0015) from the Neoarchean Carajás succession, the estimated dissolved P concentration in paleoseawater ranges from 0.004 μM (for seawater with no dissolved silica) to 0.18 μM (for seawater with 2.2 mM Si) (Table 1). The estimated concentration of 0.004 μM [P] can be regarded as a minimum but unrealistic value, given that Archean seawater was likely Si-rich (10). Concentrations reaching 0.18 μM [P] were calculated based on seawater saturated in Si with respect to amorphous silica (2.2 mM Si). However, Si content reaching 2.2 mM in seawater has been argued as a theoretical maximum for the Precambrian ocean (7). Therefore, dissolved P in Archean seawater during Carajás deposition was likely in the range of 0.004 μM < P_Carajás_ μM < 0.18 μM. The highest possible scenario for dissolved P in Carajás paleoseawater (0.18 μM), even though an overestimation, represents only 7.8% of the present-day value. This indicates a low-P content in Carajás paleoseawater compared with that in the modern ocean. It is important to note that these comparisons, as in the case of most prior works treating the Precambrian P cycle, refer to the deep ocean P reservoir that is representative of most of the global oceans in volumetric terms and is also the most likely reservoir recorded by deep-water sediments such as IF. Our estimates are in good agreement with studies advocating low P conditions during the Archean (0.04 to 0.13 μM; 7), but contrast with those arguing for P concentrations similar to (∼2.0 μM; 5) or significantly higher than (up to 100 μM; 8, 9) modern values.

In order to discuss the evolution of the marine P reservoir throughout the Archean, it is necessary to establish an approximate value of dissolved Si in ancient seawater. Based on Jones et al. (7), Si concentrations in the Archean ocean were likely between 0.5 and 1.5 mM as determined from Si:Fe ratios in IFs and Si sorption experiments to Fe-oxides. Therefore, here we used a *K*_D_ value reflecting seawater with 0.67 mM Si (*K*_D_ = 0.042), which is the proposed distribution coefficient for Archean seawater in equilibrium with respect to cristobalite, the intermediate scenario between low (0 mM) and high (2.2 mM) Si end members (Jones et al., 2015). Based on this *K*_D_ value, we estimated dissolved P using previously measured P/Fe ratios from Archean IFs (18–22, 29, 30), including previously published P compilation data sets (2, 5). We limit our discussion to Archean seawater for which marine P concentrations are highly debated and for which our new data provide important new insights, and refer the reader to other published works for an overview of sedimentary P enrichments over geological time (5, 6).

**Fig. 3.**
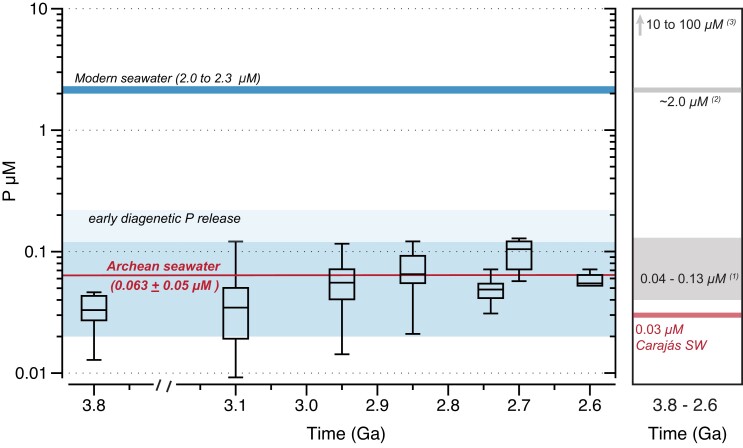
Box plot showing data distribution for estimates of dissolved phosphorus in Archean seawater with 0.67 mM of Si from 3.8 to 2.6 Ga from measured and literature P/Fe ratios (2, 16–22). Average dissolved P concentration for Archean is shown, while one standard deviation is illustrated by the shaded area. Modern seawater is shown for comparison (11). The right panel summarizes the previous estimates of P concentrations in the Archean ocean (e.g. (1) Jones et al. (7); (2) Planavsky et al. (5); (3) Rasmussen et al. (8)). The range of P concentration of Archean seawater increases when assuming that the sediments experienced a 50% loss during early diagenesis.

Based on the varying P/Fe ratios and a *K*_D_ value representative of Archean seawater, it is possible to infer that from 3.8 to 2.6 Ga, dissolved P concentrations were on average 0.063 ± 0.05 μM (*n* = 145), with minimum and maximum values of 0.01 and 0.3 μM, respectively (Fig. 3). Planavsky et al. (5) argued that Archean P levels were similar to modern-day values. However, when applying the same *K*_D_ value used in this study to their measured Archean P/Fe ratios (2.95–2.6 Ga), we estimate concentrations of dissolved P to be generally <0.2 μM, thus still supporting low-phosphorus contents in Archean seawater. We were not, however, able to reproduce higher P concentrations from 10 to 100 μM using available IF P/Fe ratio data.

Overestimations of marine P concentrations up to 100 μM during the Archean (8) seem unreasonable in light of the present results. Rasmussen et al. (8) argued that Fe-oxides did not control phosphorus removal from the water column due to the lack of correlation between P and Fe in their Paleoproterozoic data set. However, the authors did not consider that some of these correlations might not be preserved due to later diagenetic effects, such as Fe and/or P loss and/or apatite precipitation due to the reaction of vivianite and calcite associated with burial diagenesis (3). Furthermore, it may prove that special conditions (such as high alkalinity favoring P retention via apatite formation) might be required to limit postdepositional P loss and permit the preservation of adsorptive P-Fe scaling relationships in the rock record. Lastly, the authors considered the results obtained on cherts, containing virtually no Fe oxides, to be readily extrapolated to IFs (with 10 to 50 wt.% Fe), which is clearly not the case. The present data set, encompassing a large number of Archean IFs of different ages (16–22), suggests that scavenging of phosphorus by Fe-oxyhydroxide particles was the rule in Archean oceans. This further supports the convention that Fe-oxyhydroxides were indeed a primary mineral phase precipitating from Archean seawater (24). In contrast, the rare evidence available that suggests that Archean seawater P concentrations could have been relatively high (e.g. sustaining calcium phosphate mineral precipitation from seawater; 8) may possibly reflect diagenetic processes or local exceptions.

Our observation of P-Fe scaling relationships in IF consistent with partitioning via a distribution coefficient model provides new support for their application as a proxy of Archean seawater P concentrations, and our new analyses clearly indicate that Earth's ocean surface was likely P-limited. Although the present data (Fig. 1b) suggest minor modifications related to diagenesis, assuming a typical release up to 50% of the adsorbed phosphorus to the overlying seawater (7) would imply that Archean P concentrations were at least 10× lower than modern-day values (Fig. 3). This low P availability likely reflects the balance of more important phosphorous sinks (e.g. IF scavenging) relative to limited sources, such as weathering of emerged continental landmasses, which increased significantly only toward the end of the Archean (4).

## Conclusion

Our results provide evidence that scavenging of dissolved P by Fe-oxide precipitates was an active mechanism throughout the Archean Era. The concept of an Fe-P trap mechanism in the Precambrian was first suggested based on modern observational data (2, 11–13), which was later supported by numerical simulations (6, 31). However, clear empirical constraints from the rock record on marine P concentrations in deep time were lacking. IFs from Carajás, combined with a larger Archean literature data set, support the notion of a low-phosphorus ocean and highlight the capability of IFs to record past environmental conditions by the predictable nature of their trace element adsorption reactions. Using the analogous nature of P-Fe partitioning in Carajás IFs compared with modern marine environments as well as experimental studies, we estimated dissolved phosphorus contents in paleoseawater locally and globally, based on measured, experimental, and literature data. We confirm that from 3.8 to 2.6 Ga, phosphorus was most likely scarce in seawater and estimate average concentrations of 0.063 ± 0.05 μM P for this period.

This finding has implications for the P cycle in the Archean and allows us to question whether IF deposition was limiting marine phosphorus concentration. If we take the Carajás basin as an example, we can calculate the flux of P (mol yr^−1^) exiting seawater based on the average P concentration in IFs, a range of well-constrained sedimentation rates determined for IF (32, 33), and the areal extent of the basin. Considering sedimentation rates of 300, 100, and 6 m Myear^−1^, we determine a high (1.85 × 10^8^ mol year^−1^), medium (6.18 × 10^7^ mol year^−1^), and low (3.71 × 10^6^ mol year^−1^) flux of P for the Carajás basin, respectively. Given the dissolved P concentration (e.g. 0.03 μM) for Carajás paleoseawater, we can thus calculate the P amount in 100 and 1000 m depth and finally estimate a residence time for P in seawater. A maximum residence time of 146 years was calculated for the Carajás basin (i.e. considering 1,000 m depth and low flux of P), which is significantly lower than the residence time of P in the modern ocean (20,000 to 80,000 years; 34). The difference with modern values is even more distinctive if a high flux is considered (e.g. 1.85 × 10^8^ mol year^−1^), providing a residence time of 3 years. Therefore, these results imply that IFs were an important P sink and likely limited P availability in Carajás paleoseawater.

Finally, the long-term availability of phosphorus in Earth's ocean controls the capacity of oxygen to accumulate in the atmosphere by increasing primary biomass production, which will either be buried in sediments, favoring a net O_2_ accumulation, or recycled to bioavailable forms (35). Recently, it has been argued that changes in P recycling over time as a result of increased electron acceptors would have modulated biological production (36) and would have been the cause for Earth's major redox tipping point, the GOE (4). Therefore, prior to higher rates of continental weathering and corresponding P influx, and prior to the expansion of sulfidic conditions in continental margins (e.g. less effective P adsorption in sulfides; 37), phosphorous-limited seawater conditions prevailed during the first half of Earth's history and likely contributed strongly to the general anoxia of the atmosphere and ocean throughout much of the Archean Eon.

## Materials and methods

For each sample, rock powders representing 10 g or more were crushed in an agate mortar and homogenized, and aliquots of ∼250 mg were dissolved in closed teflon vessels (Savillex) at about 90°C for one day using 3 mL of concentrated HF (40%), 3 mL of concentrated HCl (32%), and 1 mL of concentrated HNO_3_ (65%). Afterward, 93 mL of H_3_BO_3_ aqueous solution (20 g L^−1^ H_3_BO_3_) was added to neutralize the excess HF. All reagents used were of analytical grade. Elements were measured by Inductively Coupled Plasma-Atomic Emission Spectrometry (ICP-AES) using a Horiba Jobin Yvon Ultima 2 spectrometer at the PSO/IUEM (Pôle Spectrométrie Océan, Institut Universitaire Européen de la Mer, Brest, France), following the analytical procedure of Cotten et al. (38). The boron included in the solution was used as an internal standard. The precision and accuracy were evaluated using international standards IF-G, ACE, JB2, and WSE. The relative standard deviation is ≤1% for SiO_2_ and ≤2% for the other major elements.

## Supplementary Material

pgad025_Supplementary_Data

## Data Availability

All data are included in the article and/or supplementary material.
