# Supplementary material for: Low-phosphorus concentrations and important ferric hydroxide scavenging in Archean seawater
Source: PNAS Nexus. 2023 Feb 8;2(3):pgad025. doi: 10.1093/pnasnexus/pgad025 (PMC10003746; doi:10.1093/pnasnexus/pgad025)
Supplement: pgad025_Supplementary_Data [file pgad025_supplementary_data.zip › PNASNEXUS-PNASNEXUS-2022-00678-T-s01.docx]

Supplementary Material: Low phosphorus concentrations and important ferric hydroxide scavenging in Archean seawater

Eric Siciliano Rego^1,2,*^; Vincent Busigny^3^; Stefan V. Lalonde^4^; Camille Rossignol^5^; Marly Babinski^1^; Pascal Philippot^2,6^

**GEOLOGICAL CONTEXT**

The Carajás Mineral Province (CMP) is located in the southeastern part of the Amazonian Craton, Brazil (Fig. S1 A), and contains remarkable metallogenic diversity including world-class deposits of iron oxide-copper-gold (IOCG), large IFs, and Mn ore (1, 2). The Carajás Basin, within the CMP (Fig. S1 B,C), comprises Neoarchean and Paleoproterozoic sedimentary units that have been deposited on a basement made up of granulites, migmatites and metavolcanic rocks emplaced from ca. 3080 Ma to ca. 3000 Ma (2–4). The crystalline basement is overlain unconformably by volcano-sedimentary sequences from the Grão Pará Group, which have undergone subgreenschist to low-greenschist facies metamorphism (4–6). At the base of the Grão Pará Group lies the Parauapebas Formation, composed by basalts and basaltic andesites, while IFs from the Carajás Formation conformably overlie or are occasionally interlayered with the basalts, a typical characteristic of Algoma-type IFs (1, 7, 8). The main lithologies comprising the Carajás Formation are oxide-facies banded iron formations alternating iron-rich and chert layers, but minor black shales and conglomeratic layers have also been reported (9, 10). These were interpreted to have been deposited in a range of environments from shallow water, low energy environments, to deep and quiet water settings (11–15).

A Late Archean to Early Paleoproterozoic sedimentary cover overlies the IFs, however its age is still debated (16). The depositional age of the Carajás IFs is well-constrained by U-Pb zircon ages of ca. 2.76-2.75 Ga in the basalts underlying and intercalating the IFs (2759 ± 2 Ma, (4); 2751 ± 4, (6)) and U-Pb zircon ages from a possible tuff (2743 ± 11 Ma) and a dolerite dyke (2740 ± 8 Ma) cross-cutting the Carajás Formation (18). The iron deposits have an average thickness of 200-250 m in the Serra Norte and 300 m in the Serra Sul regions (19). The drill core FD-55 intercepting the IF deposit in Serra Norte was selected for this study (Fig. S1 D), particularly because it has two carbonate-rich intervals intercalated with iron formations, thus reflecting changes in depositional environments (*e.g*. shallower and deeper settings).

The IF facies in core FD-55 is characterized by alternating layers of Fe-rich minerals (magnetite and hematite) and chert with microcrystalline quartz and a few Fe-carbonate (*e.g.* ankerite) disseminated in the matrix. Laminations are mm- to cm-thick and usually exhibit an homogeneous banding (Fig. S2 A). Some laminations however display wavy structures (Fig. S2 B), pinching geometries and erosion surfaces (Fig. S2 C). A gradual change is observed from IF facies to Fe-rich/IF carbonates and Fe-poor carbonates (20). The Fe-rich carbonates also comprise banded (Fig. S3 A), wavy (Fig. S3 B) and irregular Fe-oxides (mainly magnetite) layers (Fig. S3 C), while carbonate phases consist mainly of ankerite with minor calcite. Iron-poor carbonate facies show mm- to cm-thick wavy to crinkly carbonates laminae and minor iron oxides (Fig. S3 D,E). As carbonate content increases (calcite and ankerite), distinct intervals containing framboidal pyrites and organic matter were also identified (Fig. S2F, G).

**Supplementary Figure and Tables:**

**
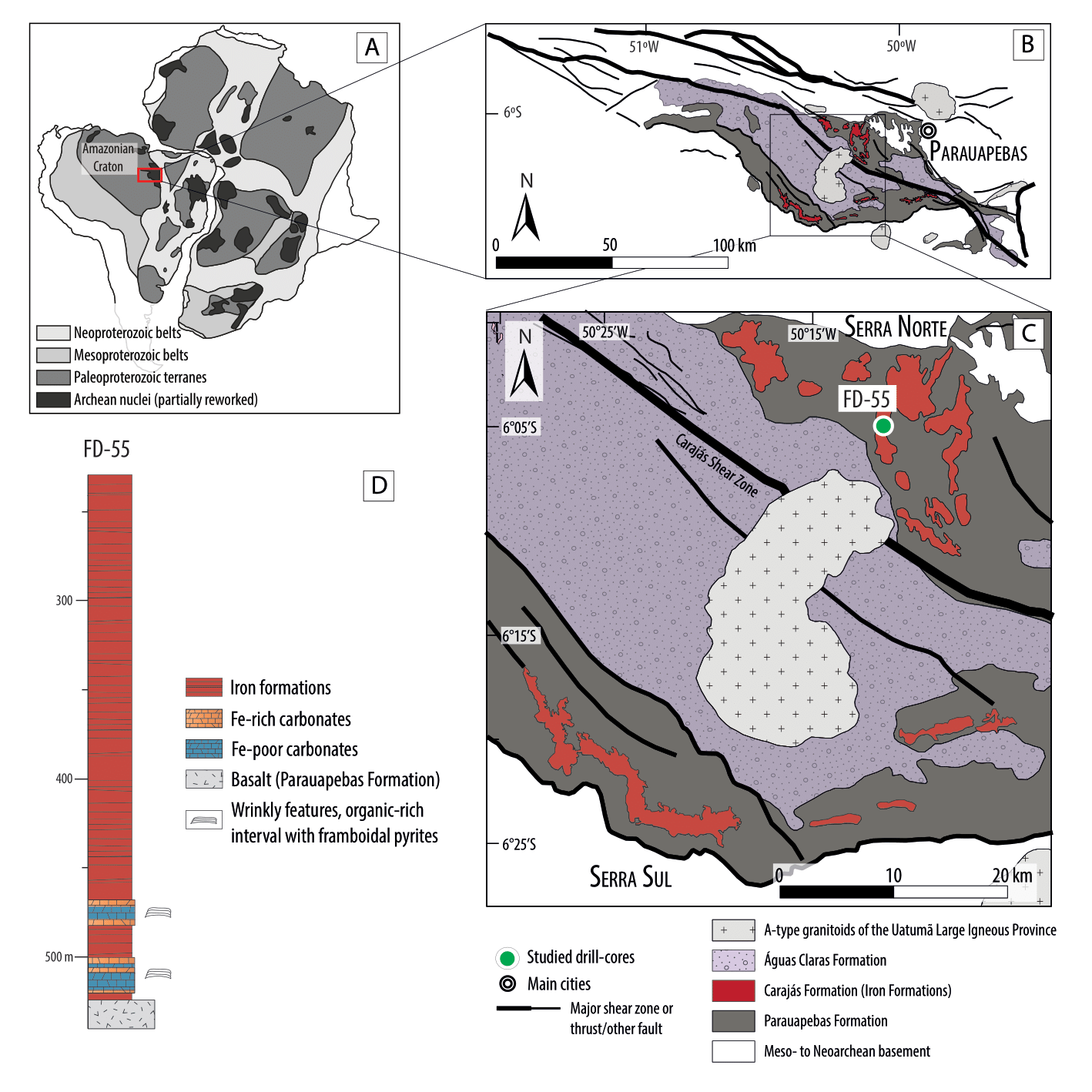
**

**Fig. S1** Geological map of the Carajás basin, located in the southeastern portion of the Amazonian Craton (A), showing the location of the drill core FD-55 in Serra Norte (B,C) and the main lithological components varying as a function of depth (m) (D) (after Rego et al., 2021).

**
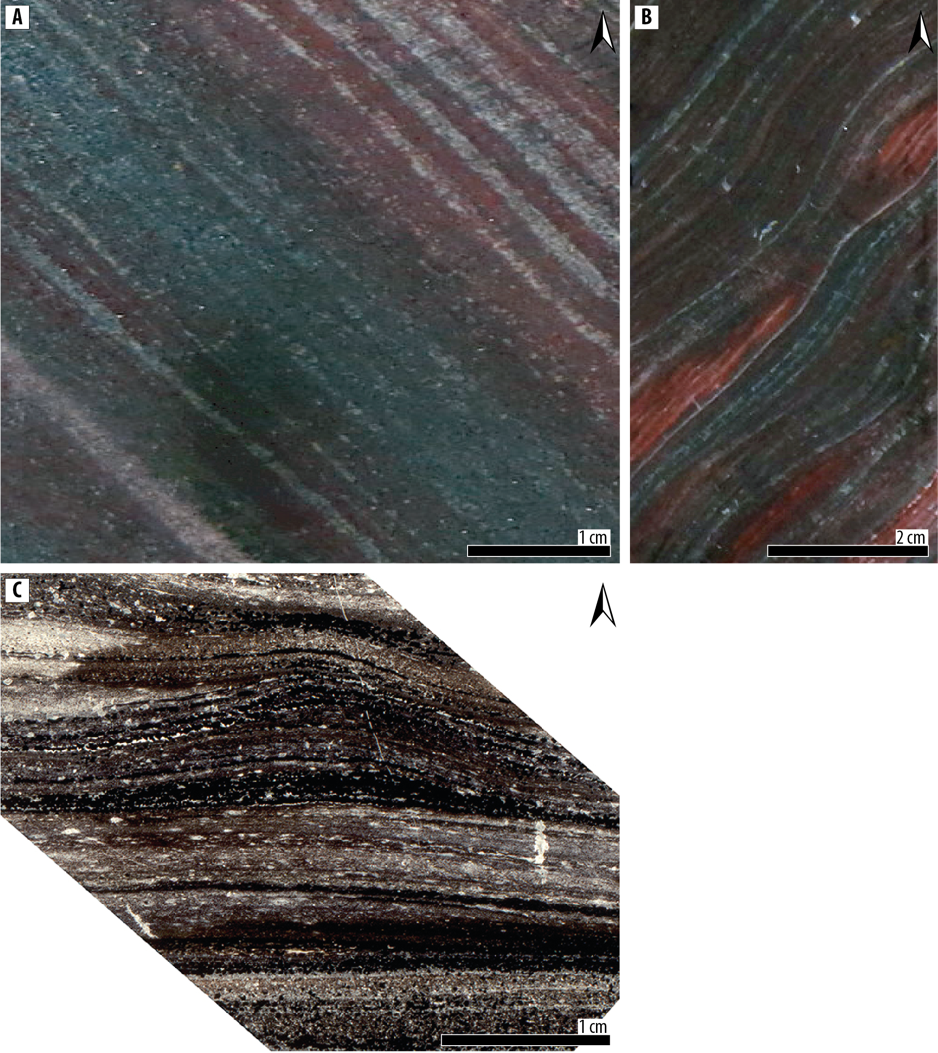
**

**Fig. S2.** Banded iron facies from the Carajás Formation. Arrows point to the stratigraphic top. **A**. Sample FD55-486.45. Fine horizontal laminations (sub-mm scale) of iron-bearing minerals (magnetite and hematite) and chert layers. **B**. Sample FD55-495.80. Finely laminated siliceous BIF displaying wavy laminations. **C.** Sample FD55-480.80. Thin section in reflected light showing a reactivation surface in a jaspilitic BIF.

**
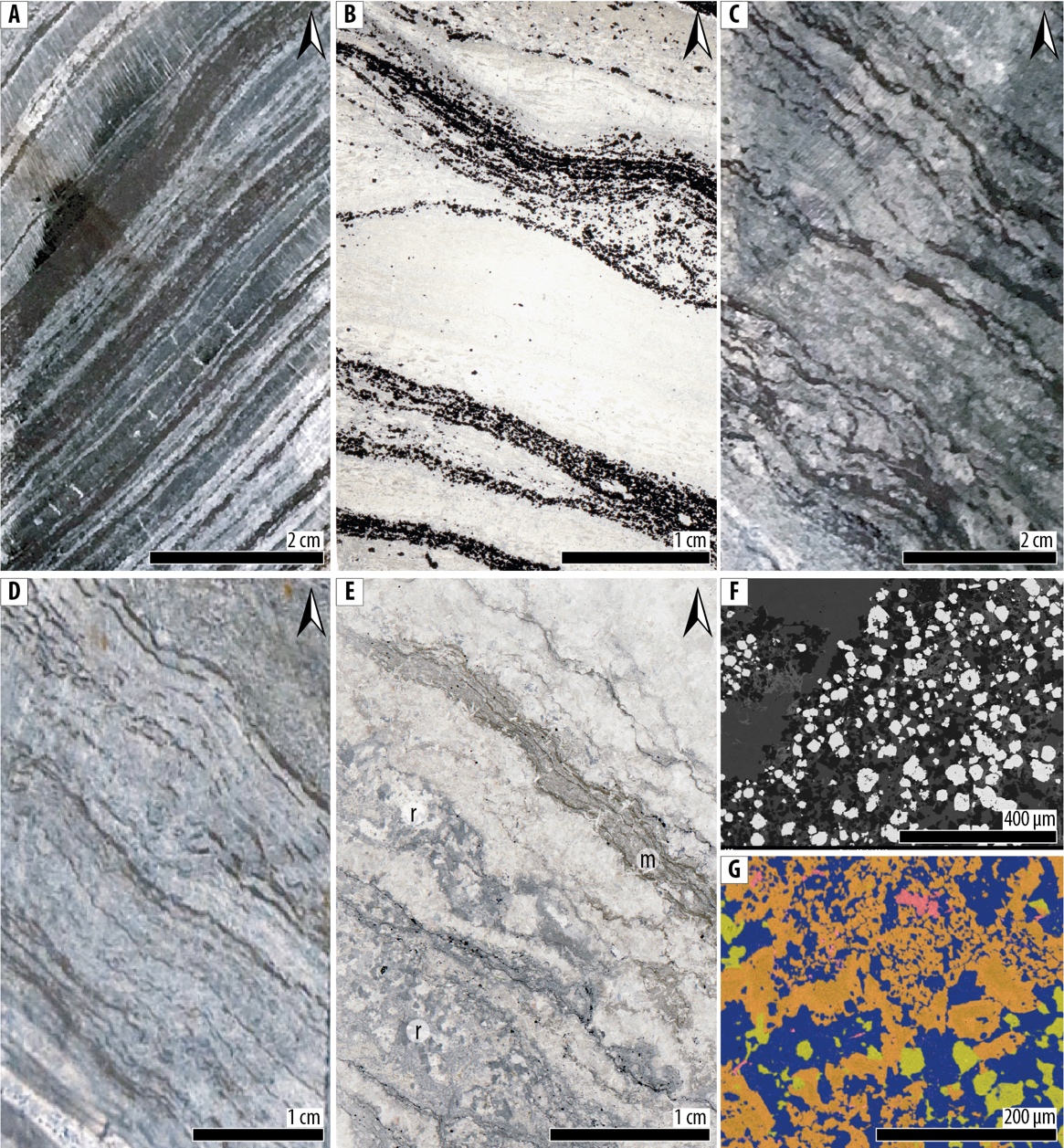
**

**Fig. S3.** Carbonate facies of the Carajás Formation. Arrows point to the stratigraphic top. **A.** Sample FD55-521.60. Fine laminations (mm to cm scale) of iron-bearing minerals (mainly magnetite) and carbonate layers. **B.** Sample FD55-521.12. Thin section in reflected light showing wavy laminations underlined by magnetite grains and carbonates. **C.** Sample FD55-518.50. Carbonate with irregular, crinkled magnetite laminations and pinching out geometries. **D.** Sample FD55-522.56. Carbonate with very fine, crinkled lamination underlined by very fine (mm thick) magnetite layers. **E.** Sample FD55-520.06. Thin section in reflected light showing a microbial mat (m) within carbonate displaying a clotted micritic texture. Some areas show recrystallized (r) textures. **F.** Sample FD55-514.57. Electron backscattered image showing diagenetic framboidal pyrite (bright white) minerals within a carbonate matrix. **G.** Sample FD55-514.57. EDS electron image showing Si, Ca and Fe-rich mineral phases.

**Fig. S4**: Variations in bulk P_2_O_5_, Al, CaO, MgO, and MnO measured in Neoarchean iron formations, Fe-rich and Fe-poor carbonates from Carajás, Brazil.


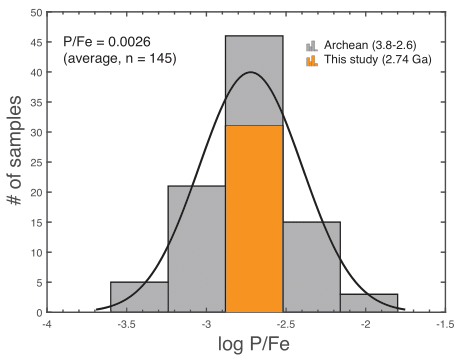


**Fig. S5.** Normal distribution shown by log P/Fe ratios from Archean and Neoarchean Carajás (this study) IFs fitted with a gaussian distribution curve.

**Table S2:** Estimations of dissolved P for Carajás samples based on K_D_ values taken from NaCl and seawater experiments, including additional K_D_ values based of experimental condition reproducing an ancient ocean with varying Si, Ca^2+^, and Mg^2+^ contents (Konhauser et al., 2007; Jones et al., 2015).

| **Experimental Conditions** | **K_D_ values** | **P (μM)^1^** |
| --- | --- | --- |
|  |  |  |
| (Konhauser et al., 2007) |  |  |
| NaCl + 0 Si | 0.075 | 0.02 |
| NaCl + 0.67 mM Si | 0.011 | 0.13 |
| NaCl + 2.2 mM Si | 0.002 | 0.73 |
|  |  |  |
| (Jones et al., 2015) |  |  |
| Seawater + 0 mM Si | 0.338 | 0.004 |
| Seawater + 0.67 mM Si | 0.042 | 0.03 |
| Seawater + 2.2 mM Si | 0.008 | 0.18 |
| 0.56 M NaCl + 0 mM Si | 0.078 | 0.02 |
| 0.56 M NaCl + 0.67 mM Si | 0.01 | 0.15 |
| 0.56 M NaCl + 0.67 mM Si + 50 mM Mg2+ | 0.054 | 0.03 |
| 0.56 M NaCl + 0.67 mM Si + 10 mM Ca2+ | 0.119 | 0.01 |
| 0.56 M NaCl + 0.67 mM Si + 50 mM Mg2+ + 10 mM Ca2+ (modern ocean [Ca and Mg]) | 0.066 | 0.02 |
| 0.56 M NaCl + 2.2 mM Si + 45 mM Mg2+ + 55 mM Ca2+ (ancient calcite sea) | 0.021 | 0.07 |
| 0.56 M NaCl + 2.2 mM Si + 10 mM Mg2+ + 24 mM Ca2+ (ancient aragonite sea) | 0.013 | 0.11 |
| 0.56 M NaCl + 0 mM Si + 50 mM Mg2+ + 10 mM Ca2+ | 0.23 | 0.006 |

^1^Estimated dissolved P based on P/Fe mol ratio (0.0015) for Carajás IFs (2.74 Ga).

**REFERENCES CITED**

1. C. Klein, E. A. Ladeira, Petrography and geochemistry of the least altered banded iron-formation of the Archean Carajás formation, northern Brazil. *Econ. Geol.* **97**, 643–651 (2002).

2. C. P. N. Moreto, *et al.*, Neoarchean and paleoproterozoic iron oxide-copper-gold events at the sossego deposit, Carajás Province, Brazil: Re-Os and U-Pb geochronological evidence. *Econ. Geol.* **110**, 809–835 (2015).

3. R. T. Pidgeon, M. J. B. MacAmbira, J. M. Lafon, Th-U-Pb isotopic systems and internal structures of complex zircons from an enderbite from the Pium Complex, Carajas Province, Brazil: Evidence for the ages of granulite facies metamorphism and the protolith of the enderbite. *Chem. Geol.* **166**, 159–171 (2000).

4. N. Machado, Z. Lindenmayer, T. E. Krogh, D. Lindenmayer, U-Pb geochronology of Archean magmatism and basement reactivation in the Carajás area, Amazon shield, Brazil. *Precambrian Res.* **49**, 329–354 (1991).

5. C. A. Rosière, *et al.*, Structure and iron mineralisation of the Carajás Province. *Trans. Institutions Min. Metall. Sect. B Appl. Earth Sci.* **115**, 126–133 (2006).

6. R. S. Krymsky, M. J. B. Macambira, J. M. Lafon, G. S. Estumano, Uranium-lead dating method at the Pará-Iso isotope geology laboratory, UFPA, Belém - Brazil. *An. Acad. Bras. Cienc.* **79**, 115–128 (2007).

7. P. L. G. Martins, *et al.*, Neoarchean magmatism in the southeastern Amazonian Craton, Brazil: Petrography, geochemistry and tectonic significance of basalts from the Carajás Basin. *Precambrian Res.* **302**, 340–357 (2017).

8. H. Dalstra, S. Guedes, Giant hydrothermal hematite deposits with Mg-Fe metasomatism: A comparison of the Carajás, Hamersley, and other iron ores. *Econ. Geol.* **99**, 1793–1800 (2004).

9. A. R. Cabral, *et al.*, Trace-element and multi-isotope geochemistry of Late-Archean black shales in the Carajás iron-ore district, Brazil. *Chem. Geol.* **362**, 91–104 (2013).

10. A. R. Cabral, *et al.*, Multiple sulfur isotopes from the Neoarchaean Serra Sul black shale, Carajás mineral province, northern Brazil. *J. South Am. Earth Sci.* **79**, 377–383 (2017).

11. G. E. Tolbert, J. W. Tremaine, G. C. Melcher, C. B. Gomes, The Recently Discovered Serra dos Carajas Iron Deposits, Northern Brazil. *Econ. Geol.* **66**, 985–994 (1971).

12. J. B. Macambira, A. Schrank, Químio-estratigrafia e evolução dos jaspilitos da Formação Carajás (PA). *Rev. Bras. Geociências* **32**, 567–578 (2002).

13. B. Ribeiro da Luz, J. K. Crowley, Morphological and chemical evidence of stromatolitic deposits in the 2.75Ga Carajás banded iron formation, Brazil. *Earth Planet. Sci. Lett.* **355**–**356**, 60–72 (2012).

14. Z. Lindenmayer, J. Laux, J. Teixeira, Consideração sobre a origem das formações ferríferas da Formação Carajás, Serra dos Carajás. *Rev. Bras. Geociências* **31**, 21–28 (2001).

15. A. P. Justo, *et al.*, Paleobasinal to band-scale REE + Y distribution in iron formations from Carajás, Amazon Craton, Brazil. *Ore Geol. Rev.* **127**, 103750 (2020).

16. C. Rossignol, *et al.*, Stratigraphy and geochronological constraints of the Serra Sul Formation (Carajás Basin, Amazonian Craton, Brazil). *Precambrian Res.* **351**, 105981 (2020).

17. A. K. Gibbs, K. R. Wirth, W. K. Hirata, W. J. Olszewski, Age and composition of the Grão Pará Group volcanics, Serra dos Carajás. *Rev. Bras. Geociências* **16**, 201–211 (1986).

18. A. F. Trendall, M. A. S. Basei, J. R. De Laeter, D. R. Nelson, SHRIMP zircon U-Pb constraints on the age of the Carajas formation, Grao Para Group, Amazon Craton. *J. South Am. Earth Sci.* **11**, 265–277 (1998).

19. V. D. R. Beisiegel, A. L. Bernardelli, N. F. Drummond, A. W. Ruff, J. W. R. Tremaine, Geologia e Recursos Minerais da Serra dos Carajás. *Rev. Bras. Geociências* **3**, 215–242 (1973).

20. E. S. Rego, *et al.*, Anoxygenic photosynthesis linked to Neoarchean iron formations in Carajás (Brazil). *Geobiology* **19**, 326–341 (2021).
